# Increasing the Therapeutic Potential of Stem Cell Therapies for Critical Limb Ischemia

**DOI:** 10.24966/srdt-2060/100024

**Published:** 2020-01-20

**Authors:** Hallie J Quiroz, Samantha F Valencia, Zhao-Jun Liu, Omaida C Velazquez

**Affiliations:** 1 Dewitt Daughtry Family Department of Surgery, University of Miami Miller School of Medicine, Miami, USA; 2 University of Miami Miller School of Medicine, Miami, USA

**Keywords:** Critical limb ischemia, Stem cell therapy

## Abstract

Peripheral Arterial Disease (PAD) is a progressive, atherosclerotic disease that at its end stage, Critical Limb Ischemia (CLI), results in severely diminished limb perfusion and causes leg pain at rest, non-healing ulcers, and tissue gangrene. Many patients with CLI fail current medical and surgical therapies and thus are deemed “no option” and require limb amputation. Novel therapies to attempt limb salvage in these “no option” patients are needed. Stem cell therapy is one therapeutic angiogenic avenue that has been tested over the last 20 years. To date, clinical trials have shown promise but with only modest improvement and none demonstrated a significant decrease in amputation rates in those treated with stem cell therapy. Thus, recent investigations into improving stem cell therapy have been the focus of our laboratory and many others. This review aims to describe recent advances in increasing the therapeutic potential of stem cell therapies for CLI.

## 3. Introduction

Peripheral Arterial Disease (PAD) is a progressive systemic atherosclerotic disease which results in the narrowing of peripheral arteries and progressively decreases extremity perfusion. The end-stage of PAD, Critical Limb Ischemia (CLI), is defined as patients with chronic ischemic rest pain, ulcers or gangrene attributable to PAD [1]. Current treatment options for PAD and CLI involve risk factor modification strategies such as smoking cessation, glycemic control (if concomitant Diabetes Mellitus), cholesterol reduction, and surgical revascularization procedures dependent upon patient specific anatomy. Unfortunately, roughly 11% of PAD patients will progress to CLI every year [2], and those with concomitant diabetes are 40 times more likely to develop CLI [3]. CLI patients have an exceedingly high 6-month mortality rate of ~20%, while another 40% require major amputation within one year of diagnosis [4]. Unfortunately, diabetic patients have a 20–30% prevalence of PAD [1,5], which is higher than that in non-diabetics and they are more likely to develop symptomatic PAD with eventual progression to CLI [6]. Diabetic patients also tend to develop multifocal disease with concomitant aberration in immune function and peripheral neuropathy, which results in poor wound healing and resultant higher amputation rates than their non-diabetic counterparts [7]. Patients who eventually require amputation despite medical therapies and surgical revascularization procedures are deemed “no-option” patients, because their disease is too advanced for current therapies to afford limb salvage. Thus, there remains an unmet need for vascular regenerative therapies to improve limb perfusion and functionality for these no-option patients to avoid amputations and potential death.

**Figure 1:**
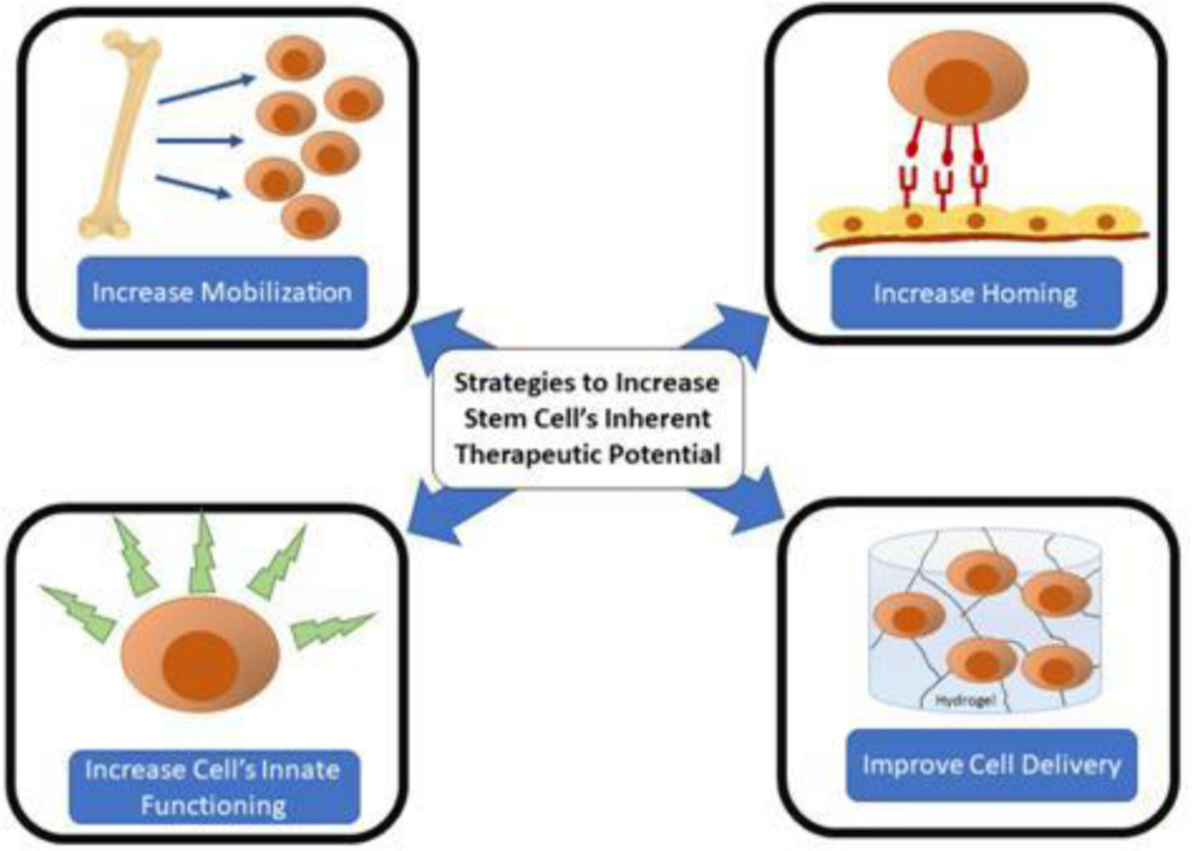
Schematic of various strategies employed in recent years to improve upon endogenous or exogenous stem cell’s inherent therapeutic potential for the treatment of limb ischemia.

Therapeutic angiogenesis, the therapeutic development of new blood vessels, may offer novel vascular regenerative strategies for limb salvage. One such strategy includes Stem Cell Therapy (SCT), and over the last two decades has resulted in over 50 phase I/II clinical trials for patients with PAD [8]. Stem cells are appealing due to their inherent ability to self-renew, differentiate into multiple cell types, exert paracrine actions (such as induce angiogenesis), and have immunomodulatory effects [9]. Necessarily the term “stem cell” is broad because it encompasses the range of embryonic stem cells, with totipotent capacity, to the adult stem cells with more limited differentiation capabilities. Mesenchymal Stem Cells (MSC) of either bone-marrow derived origin or adipose tissue-derived are by far the most commonly utilized stem cells due to their known pro-angiogenic and pro-repair phenotypes with their combined abilities to differentiate into cells required for tissue repair and regeneration in an ischemic environment [9]. Although MSC have shown promise, other stem/progenitor cells such as autologous Endothelial Progenitor Cells (EPCs) and Hematopoietic Progenitor Cells (HPC), or allogeneic stem cells derived from extra-embryonic structures are being utilized in pre-clinical and clinical trials with increased frequency, denoting their importance in angiogenic therapies [8,10–14]. Despite two decades of investigations, there currently remains a lack of finalized Phase III cell therapy trials that have shown a clinically significant decrease in the amputation free survival for treatment groups [8]. Many have suggested that the modest clinical efficacy may be due to the inherent dysfunction of host cells [11,15–18]. This review will focus on recent advances in cell-based therapies that aim to improve upon a host’s dysfunctional cells or increase the cell’s inherent therapeutic angiogenic potential (Figure 1). This may be achieved by strategies including increasing host stem/progenitor cell mobilization or homing, improve cell delivery methods, engineer cells to augment angiogenesis, utilize stem cells from extra embryonic structures, or other novel methods.

## 4. Increase Stem/Progenitor Cell Mobilization and Tissue Homing

One strategy for enhancing the therapeutic potential of stem cells is to either increase the mobilization of endogenous stem/progenitor cells or to increase stem cell tissue homing capabilities (either endogenously or via exogenously injected cells) to afford a more robust involvement in the angiogenic processes occurring in the post-ischemic environment. The augmentation of stem cell mobilization and homing as therapeutic strategies is enticing especially in patients that have been shown to have poor mobilization and homing of their stem/progenitor cells such as diabetic patients [19] and those with CLI [11]. Strategies have been described to augment their innate lack of mobilization [20]. Recently, new strategies have been developed to increase stem/progenitor cells in the circulatory system. Kwon et al. administered daily Intramuscular (IM) injections of Ac-PGP (N-acetyl Proline-Glycine-Proline, a collage-derived chemotactic tripeptide) to a murine model of hindlimb ischemia and evaluated the extent of progenitor cell mobilization (Sca1^+^Flk1^+^ cells)and the effects on ischemic limb reperfusion in a hindlimb ischemia model [21]. The same investigators had previously shown that this peptide, which is a byproduct of collagen breakdown by Matrix Metalloproteinase (MMP) and prolyl endopeptidases [22], was able to accelerate wound neovascularization and wound repair by promoting homing and engraftment of exogenous human Endothelial Progenitor Cells (EPCs) in the setting of tissue ischemia [23]. In their more recent work, IM injections of Ac-PGP improved limb reperfusion, and increased mobilization of progenitor cells [21]. Zhang et al., have also demonstrated a therapeutic increase in circulating EPCs and enhancement of their homing and angiogenic activity via the administration of recombinant growth differentiation factor-11 (rGDF11) [24]. GDF11 belongs to the Transforming Growth Factor-β (TGF-β) family and among many effects, has demonstrated protective effects against ischemic endothelial injury and atherosclerotic lesion formation [25]. Thus, Zhang et al., aimed to utilize rGDF11 to rescue EPCs from the inherent damage seen in the diabetic phenotype and have been able to demonstrate improvement in neovascularization in a rat model of diabetic limb ischemia. GDF11 restoration improved EPC mobilization and functionality, limb reperfusion, and even improved the metabolic characteristics of the diabetic rats.

Other technologies such as nanocarriers and nanodiscs have been employed to increase bone marrow mobilization and increase the homing potential of stem/progenitor cells such as MSC and EPC. Park et al. utilized nanodiscs to increase the *in vivo* stability of Substance P (SP), which is an endogenous peptide that is associated with bone marrow progenitor/stem cell mobilization [26] and induction of angiogenesis in a hindlimb ischemia model [27]. They did this by conjugating SP to HDL (high-density lipoprotein) nanodiscs, which they demonstrated had increased *in vivo* half-life and induced a more robust bone marrow mobilization response with increased therapeutic angiogenesis [28]. While this strategy focused on increasing endogenous stem cell mobilization, other investigations have focused on improving stem cell homing capabilities. Liu et al. have previously shown that E-selectin, an inducible cell-adhesion molecule, and its ligand are upregulated via Stromal-Derived Factor-1 (SDF-1α) in ischemic environments [29,30] and are required for successful neovascularization. MSC were then coated with acetylated-G5 dendrimers (Ac-G5) associated with E-selectin, known as Ac-G5-sE-sel nanocarriers. Compared to controls, these nanocarrier-coated MSCs injected systemically demonstrated superior tropism, homing, and potentiated angiogenesis and wound repair in an ischemic diabetic wound model [31]. Thus, technologies to increase stem cell mobilization and homing abilities are useful strategies that may ultimately prove useful to improve stem cell therapeutic angiogenesis (Table 1).

## 5. Increase Delivery Methods to Enhance Cell Survival, Engraftment, and Paracrine Effects

Another strategy for augmenting the therapeutic effects of stem/progenitor cell therapies is to engineer mechanisms to enhance the cell’s tissue viability. This is typically achieved by developing cell delivery vehicles that protect the cells in the microenvironment or engineering the cells via genetic modifications to enhance cell survival. Over the last couple of years, many investigators have been developing hydrogels to enhance the survival and effects of stem cells. Wang et al. developed a thermoresponsive Methyl-Cellulose (MC)-based hydrogel that allows for controlled release of placental MSC (P-MSC) over lower temperatures (T_gel_ ~ 32°C) [32], temperature of ischemic tissues has been shown to be decreased in CLI patients with chronic wounds versus healthy controls [33]. Utilization of the thermoresponsive MC-hydrogel as a vehicle for P-MSC in a hindlimb ischemia model improved tissue cell survival, limb reperfusion, and inhibited muscular atrophy [32]. Another protein-engineered hydrogel (SHIELD family) was designed at an *in situ* stiffness of 200–400 Pa (SHIELD-2.5) to protect cells from shear damages during injection, but also increased cell viability *in vitro*. When used as a vehicle for endothelial cells from induced Pluripotent Stem Cells (iPSC-EC) treatment in a hindlimb ischemia model, the SHIELD-2.5 hydrogel improved both cell tissue-retention and arteriogenesis [34]. Another type of hydrogel that has shown recent promise in other ischemic diseases are Small-Molecule (SM) hydrogels [35], that have the advantage of being inherently biocompatible, biodegradable, and are relatively easy to design and produce [36]. Thus, Huan et al. developed a SM hydrogel via a disulfide-bond reduction method to test whether P-MSC would enhance therapeutic effects in a hindlimb ischemia model [37]. Their results suggest that their novel SM hydrogel creates an artificial niche capable of increasing stem cell survival and paracrine activity, which improved the limb reperfusion and muscle regeneration [37].

Another strategy to improve bioscaffold-based tissue engineering employs the addition of peptides into the hydrogel construct to enhance stem cell survival, engraftment, and therapeutic effects. Wang et al. capitalized on this concept by constructing a Chitosan (CS) and Hyaluronic Acid (HA) based hydrogel that was immobilized with the C domain of Insulin-like Growth Factor (IGF-1C) peptide (CS-HA-IGF-1C), to determine if this engineered hydrogel could increase cell retention and paracrine effects [38]. IGF-1C was chosen as an ideal peptide due to its known ability to hasten skeletal muscle regeneration [39], improve stem cell engraftment and its resultant neovascularization [40], and protects cells against apoptosis. Thus, the investigators tested whether their engineered bioscaffold would enhance the therapeutic effects of Adipose Derived Stem Cells (ADSC) in a hindlimb ischemia model. The CS-HA-IGF-1C hydrogel demonstrated improved ADSC survival and engraftment, which promoted limb reperfusion and muscle regeneration, which the investigators ascribe to improved paracrine actions. Thus, it becomes apparent that a promising new strategy for cell-based therapies in limb ischemia is developing delivery vehicles and bioscaffolds that enhance cell survival, paracrine effects, and ultimately improve therapeutic efficacy.

## 6. Increasing Cell’s Therapeutic Potential

As mentioned previously, patients with diabetes mellitus and/or critical limb ischemia are often older and thus have older, diseased stem/progenitor cell populations as well. Thus, another strategy to improve stem cell therapeutic potential is to produce stem cells that have either been rescued from their diseased state or have been genetically engineered to enhance their pro-angiogenic/pro-repair phenotype. It has been shown that diabetic patients have impaired stem/progenitor cells when compared to healthy controls [17–19]. Thus strategies to save the therapeutic potential of these impaired cells may improve current cell therapies in patient with diabetes. Lian et al. hypothesized that mitochondrial Reactive Oxygen Species (ROS) may be elevated in diabetic patients, and thus play a role in their stem cells’ dysfunctional phenotype [41]. They demonstrated that pre-treatment of diabetic ADSC (dADSC) with a mitochondrial ROS scavenger (miTEMPO) not only increased *in vitro* differentiation and proliferation, but also improved *in vivo* angiogenic capacity [41]. Capilla-Gonzalez et al., also determined that dADSC had impaired functionality, which they demonstrated was likely due to impaired Platelet-Derived Growth Factor (PDGF) signaling. Thus, they attempted to restore the dADSC to a pro-regenerative phenotype via incubation with a recombinant form of human PDGF (PDGF-BB) and found that *in vitro* migration and proliferation were rescued in the dADSC and *in vivo* cell homing to a cutaneous injury site was improved [42]. Castilla et al., also utilized Diabetic mice (Lepr^db/db^) bone marrow cells and primed them via co-culture with SDF-1α and then utilized these pre-treated bone marrow cells to treat a diabetic wound model [10]. These cells increased wound closure rate, neovascularization, and EPC recruitment, thus demonstrating that the diabetic phenotype, which had been previously shown to have low SDF-1α levels, may be rescued from dysfunction. In addition to diabetic stem/progenitor cells having inherent dysfunction, Al Rifai et al. sought to characterize Bone Marrow Cells (BMC) from CLI patients enrolled in the Bone Marrow Autograft in Limb Ischemia (BALI) trial [43]. This trial, while efficacious in some facets of BMC therapy in CLI, did not demonstrate a difference in amputation rates between treatment groups, which is one of the main goals of cell therapies [44]. Their investigations determined that although BMC do contain MSC, they are not abundant and potentially lack pro-angiogenic capabilities. Thus, they co-cultured these MSC with an Endothelial Growth Medium (EGM)-2 and demonstrated that these Stimulated MSC (S-MSC) had improved *in vitro* proliferation and exhibited an increased transcriptome of vascular cell adhesion molecule-1 (VCAM1), which is an intermediary molecule that traffics interactions between stem/progenitor and endothelial cells, thereby increasing its homing capacity. *In vivo*, the S-MSC also demonstrated improved reperfusion in a hindlimb ischemia model and improved muscle repair [43].

Recently, another strategic therapeutic avenue is the genetic manipulation of stem cells to overexpress certain genes that may enhance angiogenic effects. This is typically achieved via vector transduction (viral or plasmid) that incorporates into the cellular DNA and results in an increased genomic expression of said gene. One such attempt by Park et al., transduced MSC via plasmid transduction to overexpress Green Fluorescent Protein (eGFP) and Vascular Endothelial Growth Factor (VEGF), they then utilized Fluorescent Activated Cell Sorting (FACS) analysis to ensure only cells expressing the target gene were included in the treatment. These were then transplanted into a hindlimb ischemia model where they demonstrated improved limb reperfusion, and muscle repair effects, when compared with control MSC [45]. Another approach, either via a Transcription Activator-Like Effector Nucleases (TALEN) [46] or lentiviral transduction [47], increased expression of dual chemotactic genes, granulocyte chemotactic protein-2 (GCP-2) and stromal-derived factor-1 (SDF-1α). Reports on stem cells with overexpression of both GCP-2 [48] and SDF-1α [10,49] have improved neovascularization in ischemic models. Thus, investigators sought to boost the therapeutic efficacy of MSC via overexpression of both GCP-2 and SDF-1α [46,47]. Both studies demonstrated that regardless of vector ( TALEN [46] or lentiviral [47]) or stem cell type (amniotic MSC [46] or adipose MSC [47]), their utilization in a hindlimb ischemia model demonstrated improved MSC angiogenic potentials *in vitro* and improved limb ischemia *in vivo* [46,47]. While most studies have attempted to alter MSC, one study performed genetic manipulations on Endothelial Progenitor Cells (EPC) to attempt enhanced therapeutic angiogenesis. Steinle et al., developed a novel method of non-integrating cell transfection via mRNAs, which would lead to transient production of the target proteins in the cell. Specifically, the proteins VEGF-A, SDF-1α, and Angiopoietin-1 (ANG-1) were transfected via mRNAs due to their known involvement in angiogenesis [50]. *In vivo*, EPC transduced with ANG-1 only were found to have the strongest angiogenic potential, however *in vitro* data suggests that the cocktail of all three mRNAs showed significantly improved tube formation. Although this study did not include a hindlimb ischemia model, this novel mRNA vector strategy with EPC is promising.

## 7. Allogeneic Stem Cells from Extra Embryonic Structures

Clearly bone marrow MSC are the most abundant stem cell type utilized for both pre-clinical and clinical trials [51], however other cell types (or other extraction locations) have been shown to be effective or more-so than MSC extracted from the bone marrow. MSC have been shown to be isolated from many locations within both the adult human (autologous) or extra embryonic (allogeneic) structures. For adult tissues these include trabecular/cortical bone, periosteum, synovial membranes, adipose tissue, tendons, skeletal muscle, peripheral blood, and bone, while extra embryonic structures containing MSC include umbilical cord blood, Wharton’s jelly, placental, and chorionic tissues. Due to the procedural risk of performing a bone marrow extraction and poor cell yield requiring *in vitro* expansion, investigations into utilizing MSCs from other locations have become increasingly popular, especially from the extra embryonic structures such as the umbilical cord blood, placental, and Wharton’s jelly as they have been shown to have increased therapeutic potentials [52,53]. One such recent pre-clinical investigation utilized an MSC-product derived from Wharton’s jelly, CardioCell, in a hindlimb ischemia model and reported improved reperfusion and leg function compared to vehicle [54]. These results have supported the initiation of a clinical trial ongoing at the writing of this text (NO CLI-Study, EudraCT number: 2016–004684-40) [55]. Multiple pre-clinical trials have also demonstrated that Umbilical Cord (UC) MSC also induce reperfusion in ischemic models [56,57], and was recently shown to have superiority over BM-MSC in a murine hindlimb ischemia model [58], and has also shown efficacy in early trials. However, these have not yet reached Phase II/III [59]. The PACE trial (NCT-03000770) Employs Placental Derived Adherent Stromal Cells (PLX-PAD) and is currently in Phase III as a double-blinded, placebo-controlled, randomized controlled trial. This “off the shelf” cell-based therapy has shown previous success in pre-clinical trials demonstrating pro-angiogenic, anti-inflammatory, and regenerative properties and in two small open label Phase I trials demonstrated favorable 1-year Amputation Free Survival (AFS) and improved pain-score and tissue perfusion. The aim of the ongoing trial utilizing PLX-PAD (NCT-03000770) is to evaluate AFS and also the tolerability and safety of this novel therapeutic approach [60].

## 8. Alternative Therapies

Although cell-base therapies have shown promise for regenerative vascular medicine, there are also concerns with their utilization. Many of these drawbacks such as poor stem/progenitor mobilization and homing, aged/diseased autologous stem cells, poor survival and engraftment, and low to modest therapeutic effects have been investigated and improved upon as this review has detailed. However, certain issues with cell therapy such as the requirement of large number of cells for therapeutic effect (thus requiring culture expansion), the potential for cancerous transformation, and possible immunologic rejection remain a concern. Thus, investigators have begun to expand both stem cell and stem-cell associated therapeutics such as utilizing induced Pluripotent Stem Cells (iPSC) and extracellular microvesicles (i.e. exosomes.) Lian et al., induced MSC differentiation from iPSCs, thus creating iMSCs. In a murine hindlimb ischemia model, the iMSC treatment was superior in both limb reperfusion and muscle regeneration when compared with control BM-MSC [61]. Similarly, exosomes from iMSCs were used as treatment in a murine hindlimb ischemia model and had higher limb reperfusion, limb salvage, and blood vessel formation [62]. Exosomes have also been successfully used for angiogenesis from ADSC [63] and have also been used alongside hydrogel delivery vehicles with improved therapeutic angiogenesis [64].

## 9. Conclusion

Although attempts at vascular regenerative medicine through stem cell therapies have not yet shown definitive and clinically pronounced improvement in amputation free survival in human clinical trials, many current pre-clinical and clinical trials are attempting to improve aspects of stem cell therapies that are believed to account for failure to produce the desired outcomes. Increasing therapeutic potential such as increasing stem/progenitor cell mobilization and homing, engineering stem cell to increase their inherent angiogenic potential, improving delivery vehicles to improve cell survival and paracrine effects, utilizing extra embryonic stem cells, and novel approaches using iPSCs and exosome therapies are the future of vascular regenerative medicine as new limb salvage treatments. As this review has demonstrated, many of these therapies have shown improved outcomes and many will likely be involved in future human clinical trials.

## Figures and Tables

**Table 1:** Summary of strategies to increase stem cell therapeutic potentials in critical limb ischemia.

Cell-Type	Methodology	Author
**Mobilization and Tissue Homing**		
Progenitor cells (Sca1^+^Flk1^+^ cells)	IM injections of Ac-PGP	Kwon et al. [23]
EPCs	IP injections of rGDF11	Zhang et al. [24]
MSCs, EPCs	IV injection of HDL-SP nanodiscs	Park et al. [28]
MSC	IV injection of Ac-G5-sE-sel nanocarrier coated MSC	Liu et al. [31]
**Improved Delivery Methods**		
P-MSC	Thermoresponsive MC-based hydrogel	Wang et al. [32]
iPSC-EC	SHIELD-2.5 hydrogel	Foster et al. [34]
P-MSC	SM-Hydrogel	Huang et al. [37]
ADSC	CS-HA-IGF-1C hydrogel	Wang et al. [38]
**Increasing Cell’s Inherent Angiogenic Potential**		
dADSC	*In vitro* co-culture of cells with miTEMPO	Lian et al. [41]
dADSC	*In vitro* co-culture of cells with PDGF-BB	Capilla-Gonzalez et al. [42]
dBMDSC	*In vitro* co-culture of cells with SDF-1α	Castilla et al. [10]
BM-MSC	*In vitro* co-culture of cells with EGM-2	Al Rifai [43]
BM-MSC	*Ex vivo* VEGF plasmid transduction	Park et al. [45]
AM-MSC	*Ex vivo* GCP-2 and SDF-1α TALEN transduction	Jeong et al. [46]
ADSC	*Ex vivo* GCP-2 and SDF-1α lentiviral transduction	Min et al. [47]
EPC	*Ex vivo* mRNA transduction of pro-angiogenic proteins	Steinle et al. [50]
**Allogeneic Stem Cells from Extra embryonic Sources**		
CardioCell	CardioCell as treatment in HLI model	Musial-Wysocka et al. [54]
UC-MSC	UC-MSC as treatment in HLI models	Pereira et al. [56] Yin et al. [57] Wang et al. [58]
PLX-PAD	Phase III trial as treatment for CLI (NCT-03006770)	Norgren et al. [60]
**Alternative Therapies**		
iMSC	Creation of MSC from iPSCs and utilizing them as treatment in HLI model	Lian et al. [61]
iMSC exosomes	iMSC exosomes as treatment in HLI model	Hu et al. [62]
ADSC exosomes	ADSC exosomes as treatment in HLI model	Figliolini et al. [63]
ADSC exosomes	ADSC exosomes coupled with hydrogel delivery method	Zhang et al, [64]

IM: Intramuscular; IP: Intraperitoneal; IV: Intravenous, MSC: Mesenchymal Stem Cells, P-MSC: Placental- MSC; BM-MSC: Bone Marrow derived MSC; UC-MSC: Umbilical Cord derived MSC; AM-MSC: Amniotic derived MSC; P-MSC: Placental MSC; ADSC: Adipose-Derived Stromal Cells; iPSC: induced Pluripotent Stem Cell; EC: Endothelial Cell; dADSC: diabetic ADSC; dBMDSC: diabetic Bone Marrow Derived Stem Cells; Ac-PGP: N-acetyl proline-glycine-proline; rGDF11: recombinant Growth Differentiation Factor-11; HDL-SP: High-Density Lipoprotein-Substance P; Ac-G5-sE-sel: Acetylated-G5 dendrimers associated with E-selectin; MC: Methyl Cellulose; SM: Small Molecule; CS-HA-IGF-1C: Chitosan, Hyaluronic Acid insulin-like growth factor-1C; PDGF-BB: recombinant Platelet-Derived Growth Factor, SDF-1α: Stromal-Derived Factor-1; EGM-2: Endothelial Growth Medium-2; VEGF: Vascular Endothelial Growth Factor; GCP-2: Granulocyte Chemotactic Protein-2, TALEN: Transcription Activator-Like Effector Nucleases, HLI: Hindlimb Ischemia, CLI: Critical Limb Ischemia, iMSC: MSC induced from iPSC.
